# Anger and Coronary Artery Disease in Women Submitted to Coronary Angiography: A 48-Month Follow-Up

**DOI:** 10.5935/abc.20180165

**Published:** 2018-09

**Authors:** Karine Elisa Schwarzer Schmidt, Alexandre Schaan de Quadros, Mauro Regis Moura, Carlos Antonio Mascia Gottschall, Márcia Moura Schmidt

**Affiliations:** Instituto de Cardiologia / Fundação Universitária de Cardiologia (IC/FUC), Porto Alegre, RS - Brazil

**Keywords:** Anger, STAXI, Personality Inventory, Coronary Artery Disease/mortality

## Abstract

**Background:**

Anger control was significantly lower in patients with coronary artery disease (CAD), regardless of traditionally known risk factors, occurrence of prior events or other anger aspects in a previous study of our research group.

**Objective:**

To assess the association between anger and CAD, its clinical course and predictors of low anger control in women submitted to coronary angiography.

**Methods:**

This is a cohort prospective study. Anger was assessed by use of Spielberger’s State-Trait Anger Expression Inventory (STAXI). Women were consecutively scheduled to undergo coronary angiography, considering CAD definition as ≥ 50% stenosis of one epicardial coronary artery.

**Results:**

During the study, 255 women were included, being divided into two groups according to their anger control average (26.99). Those with anger control below average were younger and had a family history of CAD. Patients were followed up for 48 months to verify the occurrence of major cardiovascular events.

**Conclusion:**

Women with CAD undergoing coronary angiography had lower anger control, which was associated with age and CAD family history. On clinical follow-up, event-free survival did not significantly differ between patients with anger control above or below average.

## Introduction

Cardiovascular diseases (CVD) remain the leading cause of morbidity and mortality of women in several countries, such as USA and Brazil.^1^ There are more deaths from CVD (41.3%) than from the next seven causes of death combined, and the risk of dying from CVD is six-fold greater than that from breast cancer, the major concern among women.^1^ There are sex-specific differences regarding CVD presentation, pathophysiology and clinical outcomes; however, as observed by Shivpuri S. et al.^2^, in a meta-analysis only 5 of 21 studies provided information specific to the female sex, and only a few reported sex-specific differences.^2-4^

Recent data have shown a significant increase in the incidence of cardiovascular disease and deaths among women aged 45 to 54 years, in contrast to the declining trend observed in Brazil and worldwide.^2,5^ According to the American Heart Association, women show a worse risk factor profile and higher mortality among the youngsters as compared to the elderly, in addition to high in-hospital, early and late mortality rates as compared to men.^2,6-11^ There is growing evidence that psychological factors and emotional stress, such as anger and hostility, can interfere with health behaviors and influence the onset and clinical course of ischemic heart disease.^2^ At the biological level, the expression of anger can lead to a chronic increase in the levels of catecholamines, evoking an inflammatory response, increasing interleukin-6 levels,^12^ leading to the progression of atherosclerosis, and, eventually, to the clinical manifestation of cardiovascular diseases.^13-15^

In a previous study, we have reported a significantly lower control of anger in patients with coronary artery disease (CAD), independently of the traditional risk factors, the occurrence of previous events or other aspects of anger.^16^

This study aimed at assessing the association between anger and CAD, its clinical course and predictors of low anger control in women undergoing cine coronary angiography.

## Methods

### Patients

This is a prospective cohort study. All women scheduled for elective coronary angiography because of suspected CAD during the study period were consecutively assessed. This study included women aged 18 years and older, who provided written informed consent to participate in the study. The exclusion criteria were: indication for catheterization for valvular heart disease; congenital heart disease; severe diseases with life expectancy <6 months; severe aortic stenosis; and ejection fraction <30%. The project was submitted to the Ethics Committee in Research of the institution and was in accordance with the Declaration of Helsinki and the Resolution 466/12 of the National Council of Health.

### Coronary angiography

Coronary angiography was performed according to the Judkins technique, all analyses were performed in at least two views, and the severity of the coronary obstructions was assessed by use of a digital calibration system previously validated (Siemens AxiomArtis - Munich, German). Prior to the measurements, intracoronary nitroglycerin was administered routinely at the dose of 100-200 *µ*g. Coronary artery disease was defined as ≥ 50% stenosis of at least one major epicardial artery.

### Assessment of anger

Anger is an emotional state that consists of feelings that vary in intensity from mild irritation or annoyance to intense fury and rage, and changes over time spams depending on what is perceived as injustice or frustration.^17^ Anger assessment was performed by use of Spielberger’s State-Trait Anger Expression Inventory (STAXI),^17^ a tool translated to several languages, validated in Brazil and recommended by the Federal Council of Psychology. It comprises 40 statements about the intensity of anger, how patients usually feel and how often they experience anger. Each item is rated on a four-point Likert-type scale, scored as follows: 1 for “rarely”; 2 for “sometimes”; 3 for “often”; and 4 for “almost always”. The test is subdivided into subscales: state anger, trait anger (temperament and reaction) and anger expression (anger expression-in, anger expression-out, and anger control). Trait anger is defined as a predisposition to experiencing anger, indicating lasting personality trends. It is assessed by use of questions such as: “I get angry easily”, “I get angry when my good work is not recognized”. Anger expression provides an assessment of how anger is experienced: suppression, expression or control. (Examples: “I keep things to myself”, “I do things such as slam the door”, “I boil inside, but I do not show”). As state anger assesses the amount of anger that is experienced at a particular time, that subscale was not used in the sample of in-hospital patients.

### Clinical characteristics at the beginning of the study

The clinical and socioeconomic characteristics, risk factors for CAD, previous medical history, clinical presentation of CAD and history of psychological diagnosis were assessed and included in a dedicated database. Hypertension was defined as previous diagnosis of the condition or use of anti-hypertensives. Dyslipidemia was considered present in those previously diagnosed with the condition or on lipid-lowering drugs. Diabetes mellitus was defined as the previous use of insulin or oral hypoglycemic drugs, or the presence of documented fasting blood sugar > 126 mg/dL on two occasions. Previous history of depression was defined as the occurrence of at least one major depressive episode that required treatment with antidepressants.

### Outcomes

The outcome primary cardiovascular event was a combination of cardiovascular death, acute myocardial infarction (AMI), myocardial revascularization or hospitalization due to angina. Cardiovascular death was defined as any death due to immediate cardiac causes (AMI, cardiogenic shock, arrhythmia), or death of unknown cause. Acute myocardial infarction was considered in the presence of: 1) increase and/or gradual decrease in cardiac biomarkers (preferably troponin) with at least one measure over the 99th percentile and at least one of the following criteria: 1) chest pain > 10 minutes or new ST-T changes or new left bundle-branch block; or 2) development of pathological Q waves (duration ≥ 0.03 seconds; depth ≥1 mm) in at least two contiguous precordial leads or at least two leads of adjacent limbs; or 3) evidence of viable myocardial loss or new regional wall motion abnormality on any imaging test. Myocardial revascularization comprised primary percutaneous coronary intervention (PCIp) or coronary artery bypass grafting (CABG) occurring after entrance into the study. Hospitalization due to angina was defined as hospital length of stay longer than 24 hours to assess or treat cardiac chest pain, with neither AMI nor need for myocardial revascularization.

### Follow-up

The participants were followed up for 48 months by use of visits and telephone contacts, to assess the occurrence of major cardiovascular events (MCVE), defined as cardiovascular death, AMI, myocardial revascularization (CABG or PCI) and hospitalization due to angina > 24 hours.

### Statistical analysis and justification of the sample size

The sample size was calculated with power of 80, alpha of 0.05 and 95% confidence interval. At least 250 individuals were necessary to detect a relative risk of 1.60,^18^ considering the 30% incidence of MCVE in the total group of women. Continuous variables were expressed as mean ± standard deviation, while categorical variables, as absolute number and percentage. The characteristics of the patients with CAD were compared to those of patients without CAD, using Student *t* test for independent samples for continuous variables and chi-square test for categorical variables. The women were divided into two groups according to their scores being above or below average range (26.99). Their demographic characteristics, risk factors, previous history and STAXI scores were compared by use of Student *t* test or chi-square test. Cronbach’s alpha was used to assess the internal consistency of the STAXI subscales. Kolmogorov-Smirnov test was used to assess the normality of the distribution of the scores. Multiple logistic regression analysis was used to assess the variables associated with CAD on baseline angiography and control of anger. The Kaplan-Meier curves and the log-rank test were used to compare event-free survival between patients with anger control scores above and below the sample’s average range. For all tests, a p value *<* 0.05 was considered statistically significant. All data were recorded in an Excel database for analysis with the SPSS program, version 24.0 for Windows.

## Results

From November 29, 2009, to february 3, 2010, 255 participants were included. Table 1 shows the results according to the presence of CAD, clinical history and different STAXI subscales. The patients with CAD most frequently had previous cardiac procedures (CABG and PCIp) and a lower mean level of anger control than patients without CAD, who most often were married as compared to the former. Other risk factors, previous medical history and anger subscales showed no significant differences. The multiple logistic regression analysis (Table 2) identified a relationship between CAD and low anger control, previous CABG or PCI, and marital status.

**Table 1:** Clinical characteristics, medical history and STAXI scales according to the presence of coronary artery disease (CAD)

Characteristics	CAD n = 115 (45.1%)	No CAD n = 140 (54.9%)	Total: 255 p^*^
Age (years), mean ± SD	61.0 ± 10.5	60.5 ± 9.7	0.65
White, n (%)	97 (85.1)	111 (79.9)	0.27
Married, n (%)	50 (43.5)	81 (57.9)	0.02
Schooling, years	6.2 ± 5.4	5.9 ±4.4	0.65
Current job, n (%)	26 (22.6)	28 (20.0)	0.61
Living alone, n (%)	28 (24.3)	32 (22.9)	0.78
**Risk factors**			
Hypertension, n (%)	102 (88.7)	120 (85.7)	0.48
DM, n (%)	41 (35.7)	20 (27.9)	0.18
Dyslipidemia, n (%)	*7*1 (61.7)	73 (52.1)	0.12
Smoking, n (%)	28 (24.3)	23 (16.4)	0.11
Family history of CAD, n (%)	44 (38.3)	52 (37.1)	0.85
Depression, n (%)	38 (33.0)	55 (39.3)	0.30
BMI (kg/m^2^), mean ± SD	27.6 ± 5.3	28.4 ± 6.0	0.25
**Previous medical history**			
Previous AMI, n (%)	30 (26.1)	27 (19.3)	0.19
Previous PCI, n (%)	19 (16.5)	11 (7.9)	0.03
Previous CABG, n (%)	9 (7.8)	0 (0.0)	< 0.001
**STAXI subscales**			
Trait of anger (points), mean ± SD	20.0 ± 7.9	20.7 ± 8.5	0.54
Angry temperament (points), mean ± SD	8.6 ± 4.1	8.4 ± 4.0	0.69
Angry reaction (points), mean ± SD	8.0 ± 3.5	8.6 ± 4.2	0.20
Anger expression-In (points), mean ± SD	16.03 ± 4.26	16.6 ± 5.2	0.34
Anger expression-Out (points), mean ± SD	13.2 ± 4.6	12.9 ± 4.0	0.58
Control of anger (points), mean ± SD	26.2 ± 5.00	27.7 ± 3.7	< 0.001
Anger expression (points), mean ± SD	19.0 ± 10.3	17.8 ± 9.0	0.29

SD: standard deviation; p^*^ - p≤ 0.05, Student t test or chi-square test; DM: diabetes mellitus; BMI: body mass index; AMI: acute myocardial infarction; PCI: percutaneous coronary intervention; CABG: coronary artery bypass grafting.

**Table 2:** Relationship between coronary artery disease and baseline characteristics

Characteristics	Beta Coefficient	95% CI for Beta Coefficient	Total: 255 p ^*^
Low control of anger	0.15	0.03 – 0.27	0.01
Married	- 0.12	- 0.24 – - 0.01	0.03
Previous PCI	0.14	0.04 – 0.40	0.02
Previous CABG	0.16	0.26 – 0.90	< 0.001

p ^*^ - p ≤ 0.05, Wald test; CI: confidence interval; PCI: percutaneous coronary intervention; CABG: coronary artery bypass grafting.

**Figure 1:**
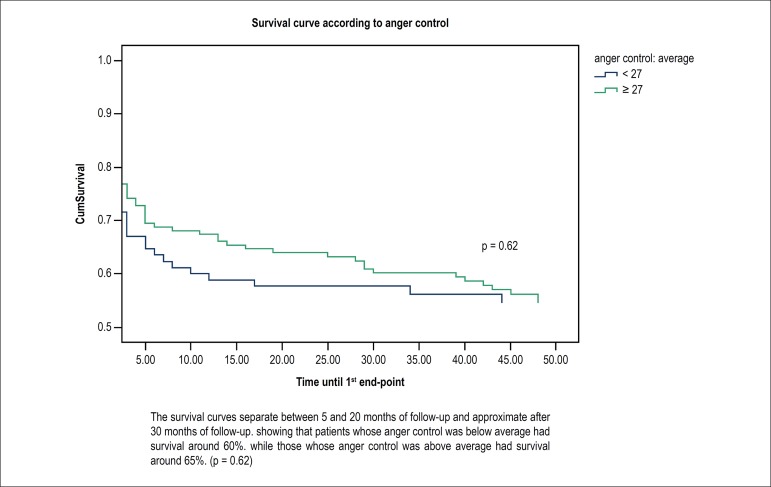
Survival curve for major cardiovascular events in up to 48 months of follow-up of women with anger control below and above average.

The patients were followed up for 48 [39-49] months to assess the occurrence of MCVE. From the initial sample of 255 patients, 10 women (3.9%) could not be reached, leaving 245 to participate in this study, 89 with anger control below the average range, and 156, over the average range. Table 3 shows the baseline characteristics of the patients regarding anger control, with 36.3% of the women with anger control below the average range and 63.7%, over the average range. Those with anger control below the average range were younger (58.1 ± 8.9 vs 62.2 ± 10.9, p < 0.001) and had a higher prevalence of family history of CAD (53.9% vs 29.5%, p < 0.001) than those whose control of anger was above the average range. Other characteristics, such as weight, diabetes, previous coronary events (AMI, PCI, CABG) and other risk factors did not differ between the two groups. However, the patients with anger control below the average range had a tendency towards lower prevalence of hypertension (p = 0.09) and previous CABG (p = 0.11). On logistic regression (Table 4), only age and the family history of CAD were predictors of poor anger control. Figure 1 shows no significant difference in event-free survival in patients with anger control below and above 27 points (p = 0.62).

**Table 3:** Patients’ clinical characteristics, medical history and STAXI scales according to anger control in a 48-month follow-up

Characteristics	Control of anger below average n = 89	Control of anger above average n = 156	Total: 245 p ^*^
Age (years), mean ± SD	58.1 ± 8.9	62.2 ± 10.9	0.001
White, n (%)	71 (79.8)	128 (83.1)	0.52
Married, n (%)	48 (53.9)	79 (50.6)	0.62
Schooling, years	6.1 ± 4.9	6.1 ± 4.9	0.97
Current job, n (%)	20 (22.5)	31 (19.9)	0.63
Living alone, n (%)	20.2 (18)	38 (24.4)	0.46
**Risk factors**			
Hypertension, n (%)	73 (82.0)	140 (89.7)	0.09
DM, n (%)	33 (37.1)	45 (28.8)	0.18
Dyslipidemia, n (%)	49 (55.1)	92 (59.0)	0.55
Smoking, n (%)	15 (16.9)	34 (21.8)	0.35
Family history of CAD, n (%)	48 (53.9)	46 (29.5)	< 0.001
Depression, n (%)	35 (39.3)	55 (35.3)	0.53
BMI (kg/m^2^), mean ± SD	28.4 ± 5.4	27.9 ± 5.9	0.55
**Previous medical history**			
Previous AMI, n (%)	22 (24.7)	34 (21.8)	0.60
Previous PCI, n (%)	8 (9.0)	22 (14.1)	0.24
Previous CABG, n (%)	1 (1.1)	8 (5.1)	0.11
**STAXI subscales**			
Trait of anger (points), mean ± SD	23.67 ± 8.25	18.52 ± 7.53	< 0.001
Angry temperament (points), mean ± SD	10.20 ± 4.05	7.51 ± 3.74	< 0.001
Angry reaction (points), mean ± SD	9.19 ± 4.04	7.81 ± 3.70	0.007
Anger expression-In (points), mean ± SD	16.85 ± 5.08	16.06 ± 4.65	0.22
Anger expression-Out (points), mean ± SD	15.07 ± 4.97	11.81 ± 3.31	< 0.001
Control of anger (points), mean ± SD	22.19 ± 3.66	29.67 ± 1.72	< 0.001
Anger expression (points), mean ± SD	25.73 ± 9.79	14.21 ± 6.74	<0.001

p^*^ - p ≤ 0.05, Student t test or chi-square test; DM: diabetes mellitus; CAD: coronary artery disease; BMI: body mass index; AMI: acute myocardial infarction; PCI: percutaneous coronary intervention; CABG: coronary artery bypass grafting.

**Table 4:** Association between control of anger and baseline characteristics

Characteristics	Beta Coefficient	95% CI for Beta Coefficient	p^*^
Age	0.15	0.002 - 0.013	0.01
Family history of CAD	0.22	0.100 - 0.340	< 0.001
Diabetes mellitus	0.007	- 0.039 - 0.170	0.21
Previous CABG	- 0.06	- 0.480 - 0.140	0.27

p ^*^ - p ≤ 0.05, Wald test; CI: confidence interval; CAD: coronary artery disease; CABG: coronary artery bypass grafting.

## Discussion

The present study identified that poor control of anger associated with CAD angiographically detected. This study found a higher percentage of married patients in the group without CAD, indicating the importance of social support in treatment adhesion and healthcare.^19^ Women with poor control of anger had a positive family history for CAD and were younger. This study corroborates that by Haukalla et al.,^20^ who reported that individuals with lower anger control were at higher risk for the first incidence of fatal and nonfatal cardiovascular disease than those who scored higher.

In this sample, the women with low anger control were younger. That characteristic can be interpreted in the sense that, as age advances, social relations are modulated through emotional regulation, which means that, as time advances and with aging, more appropriate forms of social behavior are learned, with more control over emotions and reactions.^21^ According to Cartensen’s socioemotional selectivity theory, as age advances, people become increasingly selective, tending to place a high value on positive contents and to avoid negative emotional states, because of adaptation and life changes experienced in social contexts.^22,23^

According to Shirato et al.,^24^ gender differences are evident in the success rates of the interventions to improve coronary circulation (myocardial revascularization). After a well-succeeded procedure, women submitted to coronary angioplasty had an excellent prognosis in the long run, similar to that observed in men. However, complications related to PCI and the mortality rates of women are three times higher as compared to those of men.^24^ Haukalla et al.^20^ have reported that low anger control in a 10- to 15-year clinical follow-up predicted ischemic myocardial disease in women, even after adjusting for sociodemographic variables, other cardiovascular risk factors and symptoms of depression. In the late clinical follow-up of this study, low control of anger was not associated with the occurrence of combined MCVE, myocardial revascularization included.

The literature available shows that anger is associated with several behavioral risk factors, such as tobacco use and inadequate dietary intake (hypercaloric and high sodium diets), and, in the long run, out of other cardiovascular risk factors, anger can cause LDL elevation, hypertension, diabetes mellitus, and obesity.^13,25^ In a study, Pérez-García et al.^26^ have reported that emotional discomfort and symptoms were positively associated with higher inward expression of anger and lower control of anger. In addition, they have found that preventive practices were associated with lower supression and higher control of anger, with better channeling and regulation of anger feelings. The likelihood that patients with low anger control also have low control over other risk behaviors or use them as a comfort mechanism can be considered, because the effects of well-being through neuroendocrine mechanisms of hormone release, such as serotonin, producing well-being after energetic ingestion, have been described, which would be applicable to stress/anger situations.^27^

The compensation, cognitive and affective value attributed to food overlaps the homeostatic control and the physiological signs of hunger and satiety that control food ingestion and body weight.^27,28^ However, if continuously evoked, that process would cause CAD as a factor associated not only with the feeling of anger, but with all the inappropriate coping mechanism that could accompany anger.

### Study’s forces and limitations

This study’s force resides on its female sample, because women are usually under-represented in clinical trials. This is a segment of the real world, with few losses during a long follow-up. The risk factors were assessed based on interviews with the participants, and there might have been bias of information. Assessing anger and its control, even with a tool developed for that purpose, is a hard task, considering that personality traits can be combined. Research in this area is a challenge.

## Conclusions

Women with CAD submitted to coronary angiography showed a trend towards lower control of anger, which was associated with age and the family history of CAD. The 48-month clinical follow-up showed no significant difference in the event-free time between patients with anger control scores above average range and those with anger control scores below it.
